# Inositol pyrophosphates mediate the effects of aging on bone marrow mesenchymal stem cells by inhibiting Akt signaling

**DOI:** 10.1186/scrt431

**Published:** 2014-03-26

**Authors:** Zheng Zhang, Chuanxu Zhao, Bing Liu, Dong Liang, Xing Qin, Xiujuan Li, Rongqing Zhang, Congye Li, Haichang Wang, Dongdong Sun, Feng Cao

**Affiliations:** 1 Department of Cardiology, Xijing Hospital, Fourth Military Medical University, Xi’an 710032, China; 2 Department of Cardiology, The 2nd Artillery General Hospital of Chinese People’s Liberation Army, Beijing 100088, China; 3 Department of Cardiology, People’s Liberation Army General Hospital, Beijing, China

## Abstract

**Introduction:**

Bone marrow-derived mesenchymal stem cells (BM-MSCs) have been proposed as an ideal autologous stem cell source for cell-based therapy for myocardial infarction (MI). However, decreased viability and impaired function of aged MSCs hampered the therapeutic efficacy of engrafted MSCs, and the underlying mechanisms remain unclarified. Here, we investigated the role of inositol phosphates 6 kinase (IP6Ks) inhibition on the therapeutic efficacy of BM-MSCs and its underlying mechanism.

**Methods:**

BM-MSCs isolated from young (8-week-old) or aged (18-month-old) donor male C57BL/6 mice, were subjected to hypoxia and serum deprivation (H/SD) injury with or without administration of inositol phosphates 6 kinase (IP6Ks) inhibitor TNP (10 μM). MSC apoptosis induced by H/SD was determined by flow cytometry and TUNEL assays. Protein expressions were evaluated by Western blot assay. Furthermore, the paracrine effects of MSCs were measured by reverse transcriptase–polymerized chain reaction (RT-PCR) and enzyme-linked immunosorbent assay (ELISA) analyses.

**Results:**

Aged BM-MSCs exhibited more Inositol pyrophosphate 7 (IP7) production, compared with young BM-MSCs. Meanwhile, the expression of phospho-Akt (Thr308) was significantly decreased in the aged MSCs, resulting in enhanced Bad activation and decreased Bax/Bcl-2 ratio. Moreover, the apoptosis in aged BM-MSCs was increased, compared with young BM-MSCs. Furthermore, TNP administration significantly inhibited IP7 production and increased the phosphorylation of Akt under both normoxic and hypoxic conditions. Meanwhile, IP6Ks inhibition reduced apoptotic index of aged MSCs, associated with decreased expressions of pro-apoptotic proteins Bax and Bad and increased anti-apoptotic protein Bcl-2. The expressions of angiogenic factors, including VEGF, bFGF, IGF-1 and HGF, were decreased in MSCs from aged mice. In addition, TNP administration enhanced the paracrine efficiency of aged BM-MSCs under normoxic and hypoxic conditions.

**Conclusions:**

This study demonstrates for the first time that IP6Ks and IP7 play critical role in the aging related vulnerability to hypoxic injury and impaired paracrine efficiency of BM-MSCs, which is associated with impaired Akt activation.

## Introduction

Myocardial infarction (MI) leads to permanent loss of cardiomyocytes which results in pathological remodeling [1]. Even though current therapeutic strategies can normalize coronary perfusion and, thus, ameliorate remodeling processes, the limited ability of the damaged heart to regenerate leads to congestive heart failure, which remains the leading cause of morbidity and mortality worldwide [2,3]. Recently, stem cell therapy has emerged as a promising method for treatment of ischemic heart disease [4]. Many clinical trials have demonstrated the efficacy and safety of autologous stem cells for MI treatment [5]. Among donor cell types, mesenchymal stem cells (MSCs) have been considered optimal candidates for cardiac cell therapy because of their plasticity and availability [6,7]. Indeed, intramyocardial transplantation of MSCs has been confirmed effective in treating ischemic heart disease [8,9]. However, patients suffering MI are mostly of advanced age [10]. Although the influences of aging on the properties of MSCs remain largely unexplored, aging may have significant effects on final clinical outcomes [11]. Thus, clarifying the effect of aging on MSCs and the underlying mechanisms are particularly crucial for autologous cell therapy development.

Aging is a complicated pathophysiological process accompanied by diminished activation of pro-survival kinase Akt which is correlated with greater sensitivity to hypoxic injury [12,13]. Therefore, optimizing approaches to augment impaired Akt activation and enhanced aged donor cell functional survival are crucial to optimizing cell therapy for MI.

Inositol phosphates (IPs) are a diverse group of signaling molecules that are widely distributed in mammals [14]. Most studied is inositol 1,4,5-trisphosphate (IP3), which regulates cellular calcium. 5-Diphosphoinositol pentakisphosphate (5-[PP]-IP5 or IP7), yielded from inositol hexakisphosphate (IP6) phosphorylated by inositol hexakisphosphate kinases (IP6Ks), is found to serve multiple biological functions including apoptosis and insulin secretion [15,16]. Recent studies indicate that IP7 synthesized by IP6K appears to inhibit Akt [17]. Moreover, N6-(p-nitrobenzyl) purine (TNP), a purine analogue, is identified as an inhibitor of IP6Ks *in vitro* which inhibited inositol pyrophosphate 7 (InsP7) synthesis without affecting the production of other inositol phosphates [18]. Therefore, TNP is a useful pharmacological tool to further understand the role of InsP7 synthesis in the aging process of MSCs. Accordingly, we hypothesized that IP7 plays a critical role in impaired Akt activation which contributes to the poor viability and function of aged MSCs. Furthermore, age-related IP7 synthesis may be a promising novel target for augmenting the MSC therapeutic efficacy for MI.

## Methods

### Animals

Young (8-week-old) and aged (18-month-old) male C57BL/6 mice were used for the isolation of bone marrow mesenchymal stem cells (BM-MSCs) as described previously [11]. All procedures were approved by the Animal Care and Use Committee of the Fourth Military Medical University (Approval ID: 12119) and were in compliance with Guidelines for the Care and Use of Laboratory Animals, as published by the National Academy Press.

### Isolation, culture and characterization of mesenchymal stem cells

BM-MSCs were isolated using a standard protocol as described previously [7]. Briefly, femoral and tibia marrow were isolated and flushed with phosphate-buffered saline (PBS). The adherent MSC were propagated and maintained in high glucose (Dulbecco’s) modified Eagle’s medium ((D)MEM) supplemented with 10% fetal bovine serum (FBS) and 1% penicillin/streptomycin medium. Third-passage MSCs were used for experiments to avoid contamination with other cell types.

MSCs were characterized by flow cytometry for surface markers expression and *in vitro* differentiation as previously reported [7]. In brief, after being incubated with monoclonal phycoerythrin (PE)-conjugated antibodies against CD markers (BD, San Jose, CA, USA) for one hour, BM-MSCs isolated from young and aged mice were processed through a FACS Caliper system (BD) according to the manufacturer’s protocol. For *in vitro* differentiation, BM-MSCs were induced with adipogenic media (αMEM with 10% FCS, 1% antibiotics, 50 μM indomethacin (Sigma, Saint Louis, Missouri, USA), 0.5 mM IBMX (Sigma) and 1 μM dexamethasone) and osteogenic medium (OM, 10% FBS, 0.1 μM dexamethasone, 10 mM β-glycerophosphate, and 0.2 mM ascorbic acid in α-MEM) for 21 days, which was estimated by Oil red O and alizarin red S stain, respectively.

### Hypoxia/serum deprivation injury

After IP6Ks inhibition with TNP (10 μM for two hours), MSCs were stimulated with hypoxia/serum deprivation (H/SD) injury as described previously [7]. Briefly, after being replaced in Hanks buffer, BM-MSCs were exposed to hypoxia (94% N_2_–5% CO_2_–1% O_2_) in an anaerobic system (Thermo Forma, Waltham, Massachusetts, USA) at 37°C for six hours. In the control group, MSCs were maintained at normoxia (95% air–5% CO_2_) for equivalent periods.

### Inositol polyphosphates analysis

To test the concentration of IP7 in MSCs, we monitored inositol polyphosphate levels by HPLC after labeling with [^3^H]-inositol [16]. In brief, MSCs were seeded onto 24-well plates at 1 × 10^6^ cells/ml. After washing with inositol free medium, cells were cultured with 100 μCi/ml myo- [^3^H]inositol in (D)MEM supplemented with 10% FBS for 72 hours. Then, cells were scrapped after being lysed with 0.5 M trichloroacetic acid. The supernatant was collected and prepared for HPLC analysis. The inositol phosphates were eluted by HPLC with an optimal gradient.

### Flow cytometry assay for MSCs apoptosis

MSC apoptosis induced by H/SD was determined by flow cytometry assay using an Annexin V-FITC/PI Kit (Merck, Whitehouse Station, New Jersey, USA) according to the manufacturer’s instructions [7]. Briefly, MSCs were harvested with 0.025% trypsin (Sigma Aldrich, Saint Louis, MO, USA) and incubated with 10 μl of Annexin V solution and 5 μl propidium iodide (PI) for 30 minutes at room temperature. The apoptosis of MSCs was analyzed on a FACSC-LSR (Becton, Dickinson and Company, San Jose, CA, USA).

### Terminal deoxynucleotidy1 transferase-mediated dUTP nick end-labeling (TUNEL) assay

To confirm the apoptosis of MSCs, *in situ* detection of DNA fragmentation was performed using the TUNEL assay with an assay kit (In Situ Cell Death Detection Kit; Roche Diagnostics, Basel, Switzerland) according to the manufacturer’s instructions [19]. Briefly, after H/SD injury, MSCs were incubated with terminal deoxynucleotidy1 transferase (TdT) and fluorescein-labeled dUTP for 45 minutes at 37°C followed by 4,6-diamidino-2-phenylindole (DAPI) for the identification of the nucleus. The percentage of apoptotic cells was termed the apoptotic index. All of these assays were performed in a blinded manner.

### Caspase-3 activity measurement

Caspase-3 activity was measured with the Caspase-3 Assay kit (Clontech, Mountain View, California, USA) according to the manufacturer’s instructions. Briefly, substrate cleavage was monitored fluorometrically with a SpectraMax Gemini spectrophotometer (Molecular Devices, Sunnyvale, California, USA) with excitation and emission wavelengths of 350 nm and 450 nm.

### Western blot assay

The expressions of Akt, Bax, Bcl-2 and Bad in MSCs were assessed by Western blotting following standard protocol [20]. Briefly, MSCs were lysed at 4°C in buffer containing Tris-buffered saline, 0.1% Triton X-100 (1 mM, Sigma), 4% glycerol, ethylenediaminetetraacetic acid (EDTA) (1 mM, Sigma) and protease inhibitor phenylmethanesulfonyl fluoride (PMSF) (1 mM, Roche Molecular Biochemicals). The insoluble material was removed by centrifugation at 6,000 × g, and the supernatants were diluted in SDS sample buffer. Equal amounts of protein (50 μg/lane) were separated by electrophoresis on 12% SDS-PAGE gels for 90 minutes at 120 V and sequentially electrophoretically transferred to nitrocellulose (NC) membranes. After blocking, NC membranes were subjected to immunoblotting with primary antibodies against Akt (1:500), p-Akt (1:500), p-Bad (1:500), Bad (1:500), Bcl-2 (1:500), Bax (1:500) and β-actin (1:2000) over night at 4°C. After incubation with the appropriate secondary antibody conjugated with horseradish peroxidase, blot bands were visualized with an enhanced chemiluminescene system (Amersham Bioscience, Fairfield, Connecticut, USA). Densitometric analysis of Western blots was performed using VisionWorks LS, version 6.7.1.

### Evaluation of VEGF, bFGF, IGF-1 and HGF

After H/SD injury, MSCs were harvested and analyzed by RT-PCR for vascular endothelial growth factor (VEGF), basic fibroblast growth factor (bFGF), insulin-like growth factor-1 (IGF-1) and hepatocyte growth factor (HGF). RT-PCR conditions were standard for all samples. Briefly, total cellular RNA was extracted by RNeasy (QIAGEN, Hilden, Germany) and was reverse transcribed using the Transcriptor First Strand cDNA Synthesis Kit (Roche Molecular Biochemicals, Mannheim, Germany) according to the manufacturer’s instructions. The samples were subjected to 40 cycles of amplification at 95°C for 15 seconds followed by 64°C for 20 seconds and 72°C for 25 seconds by using the specific primers. The results of the assays were normalized to the level of GAPDH.

The concentrations of VEGF, bFGF, IGF-1 and HGF secreted by MSCs were also assessed by ELISA (Life Technologies, Carlsbad, California, USA) according to the manufacturer’s instructions as previously described [20]. All samples and standards were measured in duplicate.

### Statistical analysis

The results are presented as mean ± standard error of the mean (SEM). Statistics were calculated using Prism 5.0 (GraphPad Software Inc, San Diego, CA, USA). Statistical comparisons for different groups were performed using either the Student’s *t* test or one-way analysis of variance (ANOVA). *P* values <0.05 were considered statistically significant.

## Results

### Characterization of MSCs

**Figure 1:**
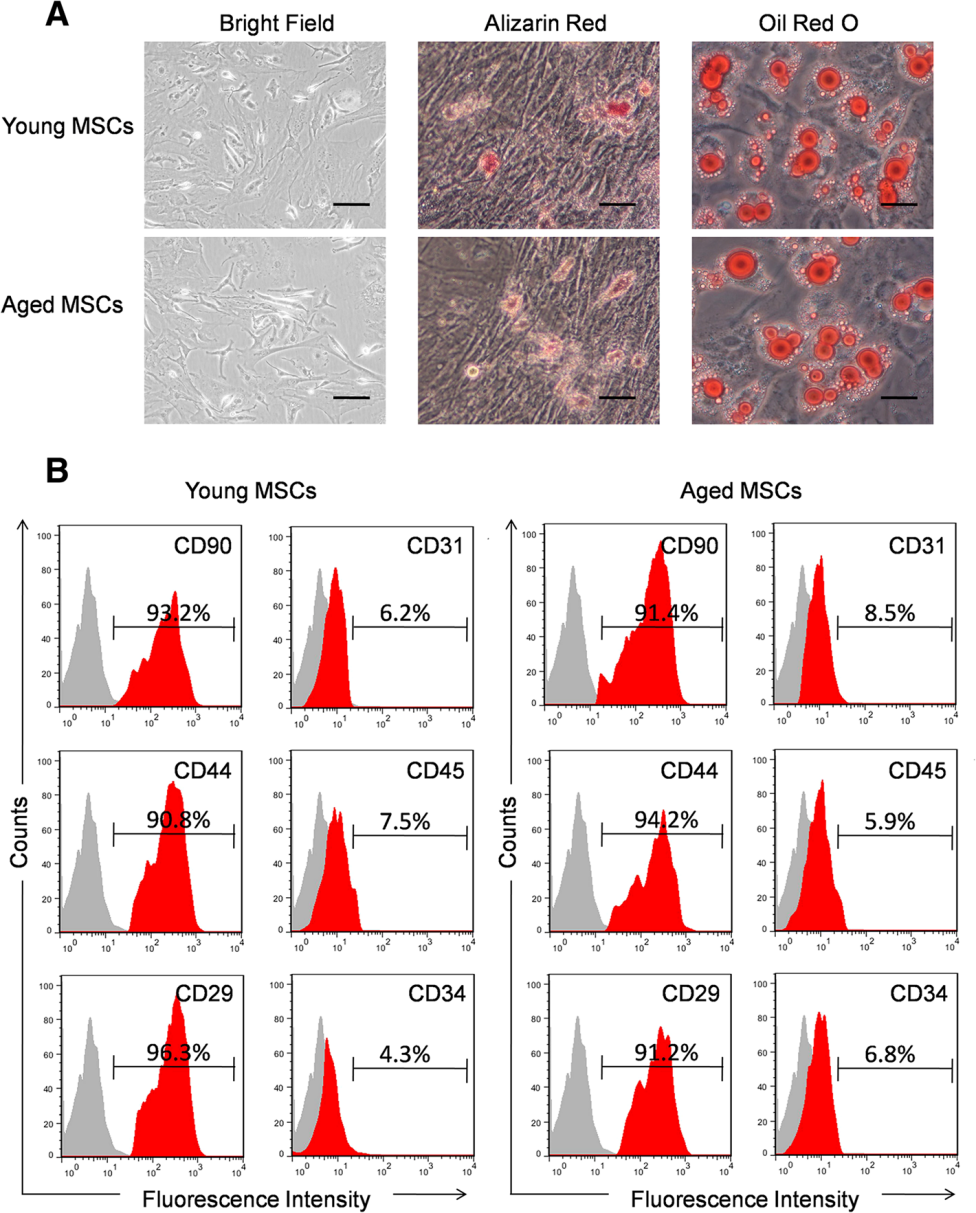
**Characterization of mesenchymal stem cells from both aged and young mice. (A)** MSCs from both young and aged donor mice display fibroblast-like morphology. The pluripotency of MSCs from both young and aged mice was confirmed by adipogenic and osteogenic differentiation assays which were detected by Oil red O staining and alizarin red S staining, respectively. **(B)** Flow cytometry results show that MSCs from both young and aged donor mice were uniformly negative for CD34 and CD45, and positive for CD44 and CD90 (n = 5). MSCs, mesenchymal stem cells.

The multipotency of MSCs from both young and aged mice was confirmed by adipogenic and osteogenic differentiation assays. After being induced with adipogenic media for 21 days, approximately 80% of MSCs had an adipocyte phenotype which was assessed by Oil red O staining. To examine the osteoblastogenic differentiation ability of MSC, cells were incubated in osteoblastogenic medium and stained by alizarin red S, demonstrating calcium deposits (Figure 1A). Flow cytometry results revealed that MSCs from both young and older donor mice expressed the MSC markers CD90, CD44 and CD29, while they were negative for the haematopoietic markers CD31, CD34 and leukocyte common antigen CD45 (Figure 1B).

### Aged mesenchymal stem cells secreted more IP7

**Figure 2:**
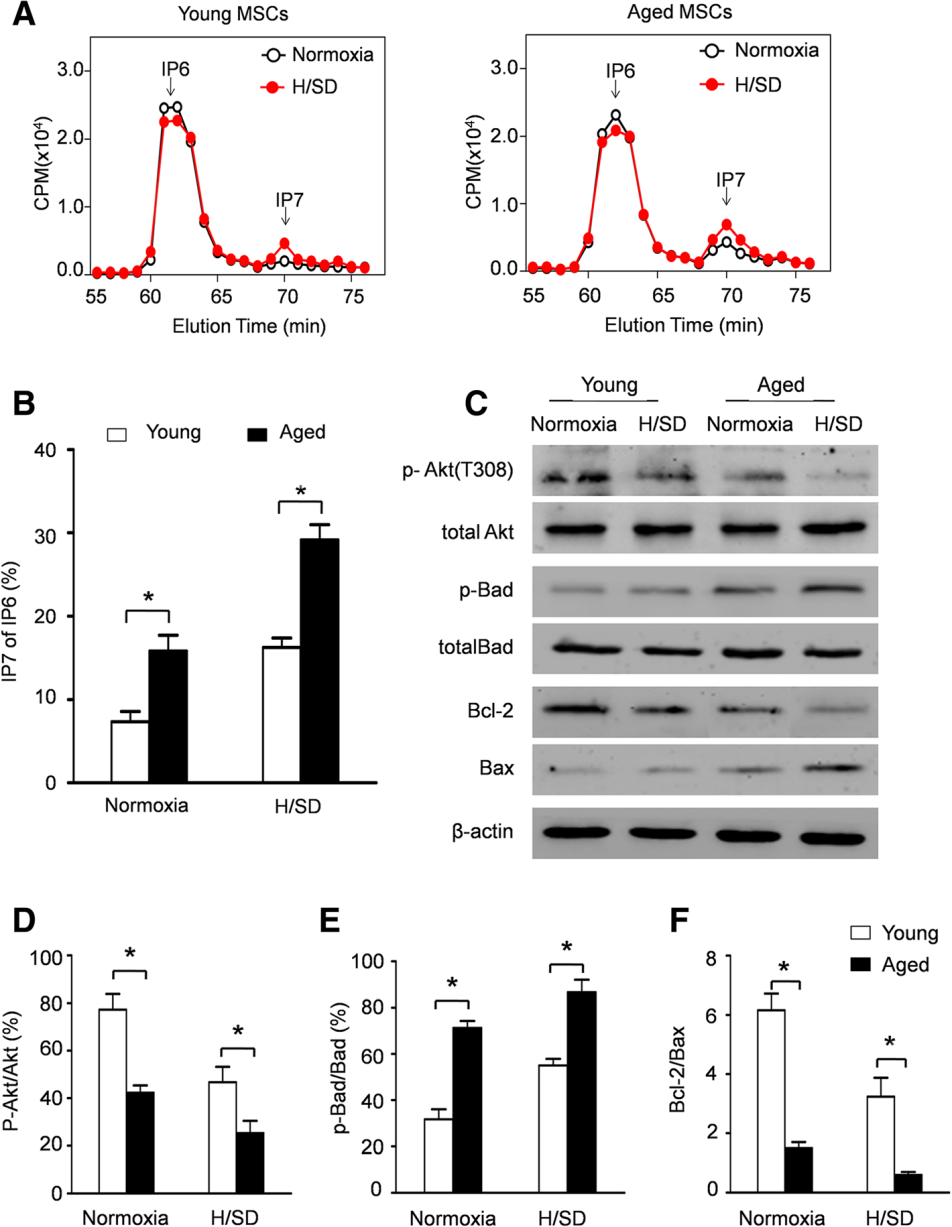
**IP7 synthesis and Akt activation in young and aged MSCs after hypoxia and serum deprivation (H/SD) injury. (A)** HPLC profiles of inositol phosphates isolated from young and aged MSCs under normoxic or hypoxic conditions. **(B)** Quantitative analysis of IP7 production as a percentage of IP6 in MSCs. **(C)** Representative Western blots of *p*-Akt (T308), Akt, *p*-Bad, Bad, Bcl-2 and Bax expression in young and aged MSCs after H/SD. Semiquantitative analysis of *p*-Akt **(D)**, *p*-Bad **(E)**, Bcl-2 and Bax **(F)**. Data are expressed as mean ± SEM. n = 5, *P <0.05, CPM, counts per minute. H/SD, hypoxia and serum deprivation; IP7, 5-diphosphoinositol pentakisphosphate; MSCs, mesenchymal stem cells; SEM, standard error of the mean.

To evaluate the synthesis of IP7in MSCs, we monitored inositol polyphosphate levels by HPLC (Figure 2A,B). There was no difference in the IP6 expression in both groups. However, in comparison to young MSCs, increased IP7 synthesis was observed in aged MSCs under normoxic condition (15.83 ± 1.09% of IP6 versus 7.45 ± 0.73% of IP6, *P* <0.05). Notably, H/SD injury further promoted IP7 synthesis in aged MSCs (29.16 ± 1.04% of IP6 versus 16.25 ± 0.66% of IP6 in young MSCs, *P* <0.05).

### Aging was associated with decreased Akt activation

Akt is an upstream regulator of the Bcl-2 family which regulates cell apoptosis and proliferation. To determine the effect of aging on the activation of Akt signaling, phosphorylated Akt in MSCs of young and aged mice was determined by Western blotting. As shown in Figure 2C-D, the expression of phospho-Akt was significantly decreased in the aged MSCs, indicating impaired Akt activation in aged MSCs. Moreover, H/SD injury further deteriorated aging-associated hyp-phosphorylation of Akt in MSCs. Compared with young MSCs, enhanced expression of the pro-apoptotic protein Bad and decreased Bax/Bcl-2 ratio were also observed in aged MSCs (Figure 2C,E,F).

### Aging increased the apoptosis of MSCs induced by H/SD injury

**Figure 3:**
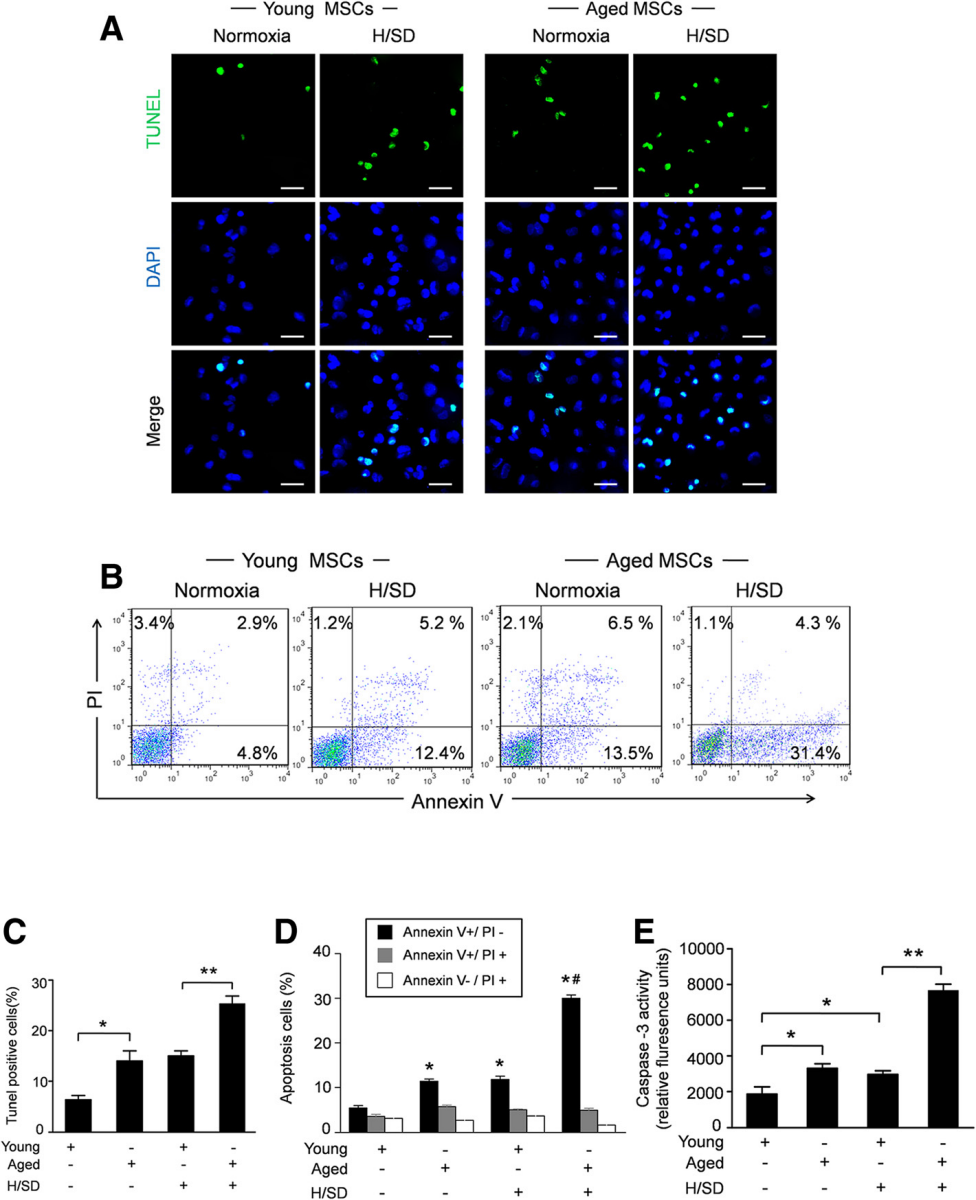
**Aging increased the apoptosis of MSCs induced by H/SD injury. (A)** Representative immunofluorescence images of apoptosis (TUNEL, green fluorescent) and DAPI (blue fluorescence) in young and aged MSCs under hypoxic or normoxic conditions (Scale bars: 50 μm). **(B)** Apoptosis was also analyzed by flow cytometry after staining with Annexin V and propidium iodide (PI). **(C)** Quantification of the apoptotic MSCs is presented as the percentage of apoptotic cells. **(D)** Quantification of the FACS analysis. Viable cells: Annexin V^-^/PI^-^; early apoptosis: Annexin V^+^/PI^-^; late apoptosis: V^+^/PI^+^; necrotic: V^-^/PI^+^. **(E)**: Caspase-3 activity of MSCs in all groups. Data expressed as mean ± SEM. n = 5, *P <0.05, #*P* <0.05 versus young + H/SD. DAPI, 4,6-diamidino-2-phenylindole; FACS, fluorescence-activated cell sorting; H/SD, hypoxia and serum deprivation; MSC, mesenchymal stem cells; SEM, standard error of the mean; TUNEL, terminal- deoxynucleotidyl transferase mediated nick end labeling.

To analyze the effect of aging on the apoptosis of MSCs, TUNEL assay and flow cytometry assays were performed. As representative photomicrographs show in Figure 3A, TUNEL-positive MSCs were observed more frequently in aged MSCs. Quantitative analyses revealed that the apoptosis index in aged MSCs was 14.03% ± 1.5% under normoxic conditions and 26.27% ± 1.4% under hypoxic conditions, significantly higher than that in young MSCs (6.33% ± 0.5% under normoxic conditions, 15.01% ± 0.6% under hypoxic conditions, Figure 3C). Representative flow cytometry results in Figure 3B revealed significantly enhanced apoptosis of aged MSCs (10.66 ± 0.55% versus 6.14 ± 0.61% in young MSCs, *P* <0.05). Moreover, aging further increased the apoptosis of MSCs induced by hypoxia (24.32 ± 0.65% in aged MSCs versus 13.24 ± 0.51% in young MSCs, *P* <0.05, Figure 3D). Concurrently, aging also significantly increased the caspase-3 enzymatic activity in MSCs under normoxic or hypoxic conditions (P <0.05, Figure 3E). These data suggest that aging increases the apoptosis of MSCs under both normoxic and hypoxic conditions.

### IP7 hampered Akt phosphorylation in aged MSCs

**Figure 4:**
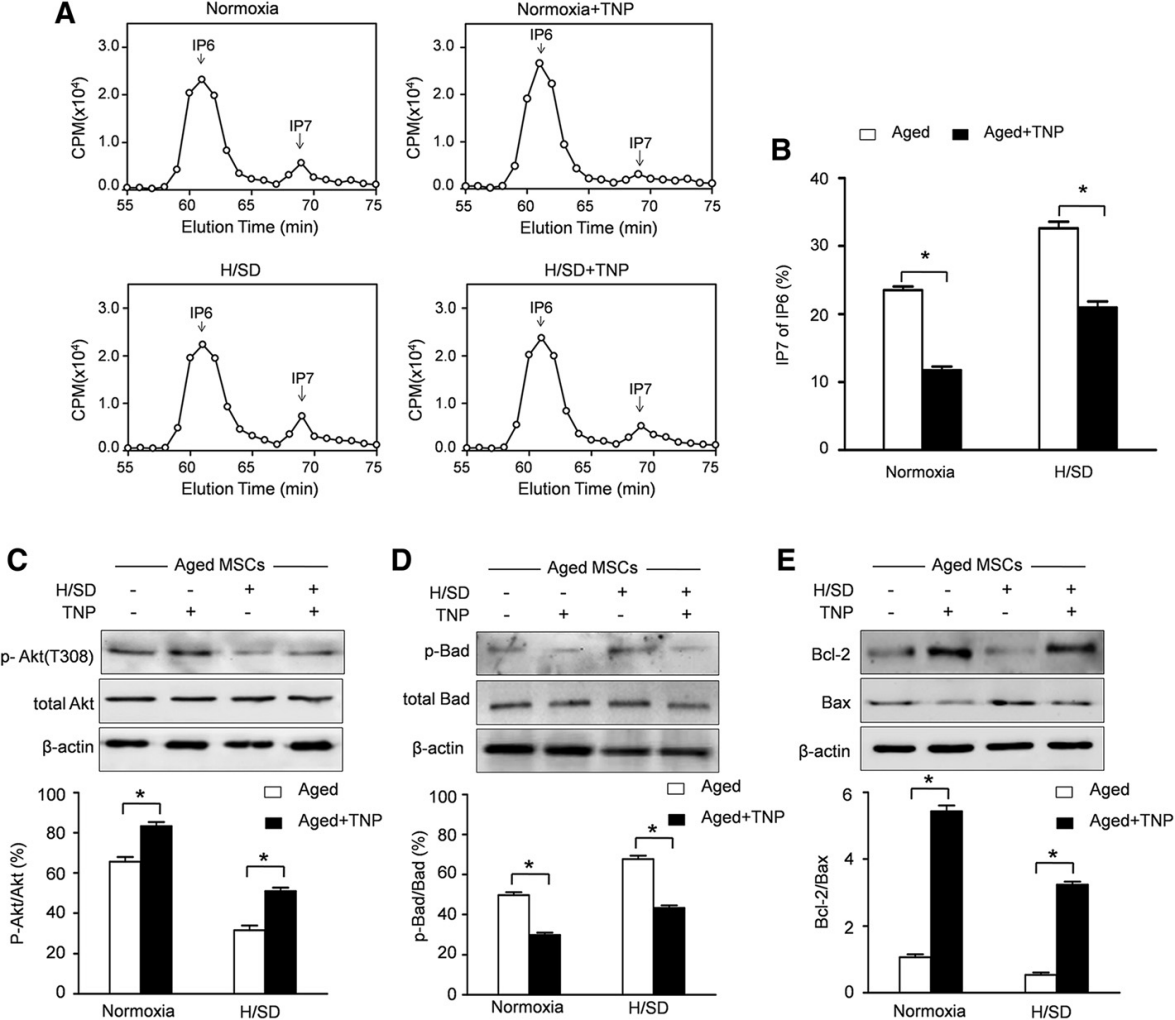
**TNP decreased IP7 production and enhanced Akt activation in aged MSCs. (A)** HPLC profiles of inositol phosphates isolated from aged MSCs with or without the IP6Ks inhibitor TNP. **(B)** Quantitative analysis of IP7 production as a percentage of IP6 in MSCs. Representative Western blots and semiquantitative analysis of the expressions of *p*-Akt (T308) **(C)**, *p*-Bad **(D)**, Bcl-2 and Bax **(E)** in aged MSCs after H/SD with or without TNP incubation. Data are expressed as mean ± SEM. n = 5, *P <0.05. CPM, counts per minute. H/SD, hypoxia and serum deprivation; IP6Ks, inositol hexakis phosphate kinases; IP7, 5-diphosphoinositol pentakisphosphate; MSCs, mesenchymal stem cells; SEM, standard error of the mean; TNP, N6-(p-nitrobenzyl) purine.

After being incubated with TNP, a selective inhibitor of IP6Ks, IP7 production in aged MSCs was significantly hampered both under normoxic and hypoxic conditions (Figure 4A,B). Notably, inhibition of IP6Ks further promoted the phosphorylation of Akt in aged MSCs (Figure 4C). Moreover, the decreased expressions of the pro-apoptosis proteins Bax and Bad were observed in aged MSCs with TNP pretreatment. Furthermore, TNP increased the expression of the anti-apoptosis protein Bcl-2 (Figure 4D,E). These data indicate that increased IP7 synthesis hampers Akt activation in aged MSCs.

### IP6Ks inhibition prevented apoptosis of aged MSCs

**Figure 5:**
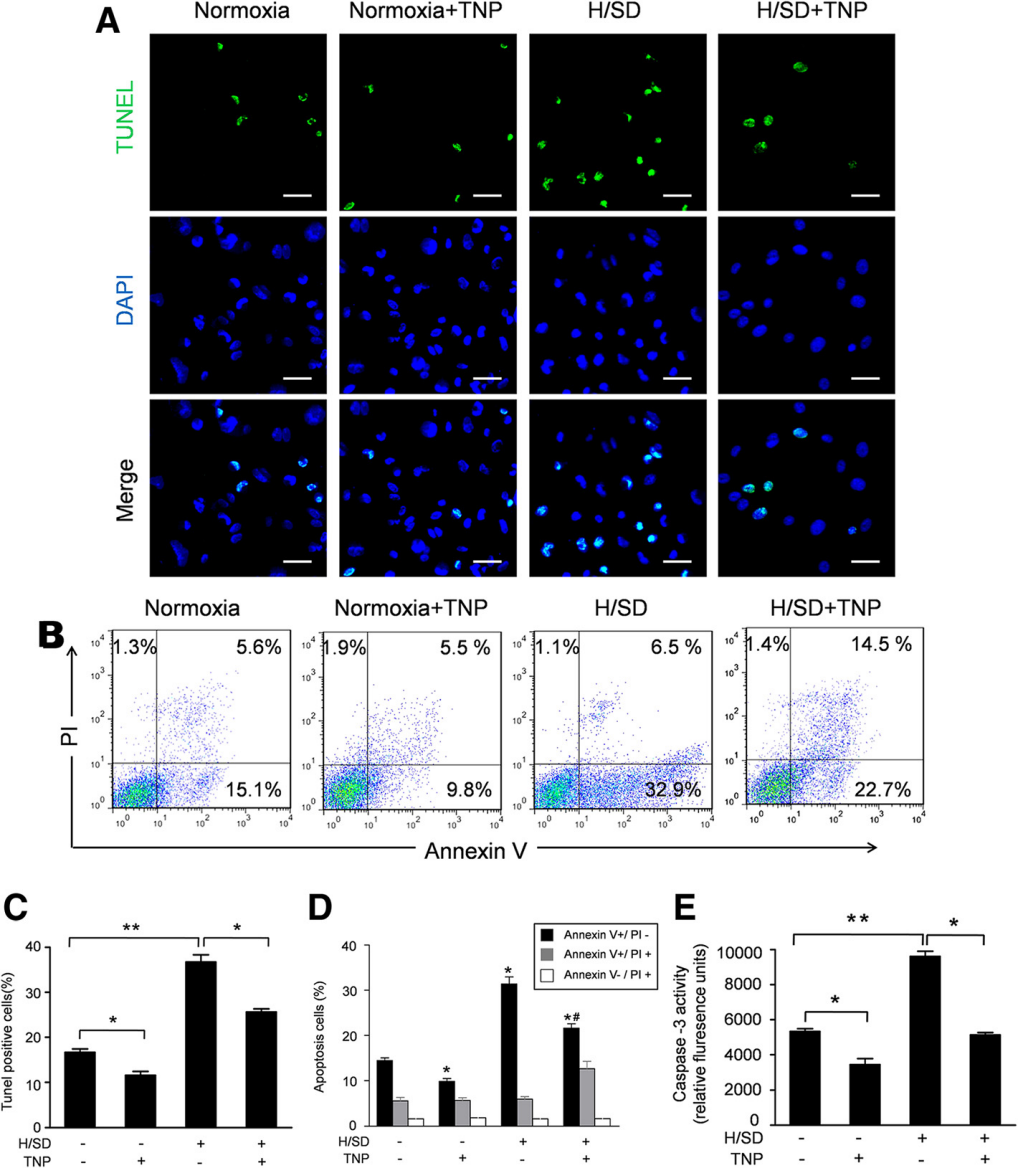
**TNP decreased apoptosis of aged MSCs. (A)** Representative confocal microscopic images of TUNEL stains for the hypoxia-induced apoptosis in aged MSCs with or without the IP6Ks inhibitor TNP. **(B)** Apoptosis was also analyzed by flow cytometry after staining with Annexin V and PI. **(C)** Quantification of the TUNEL assay is presented as the percentage of apoptotic cells. **(D)** Quantification of the FACS analysis. Viable cells: Annexin V^-^/PI^-^; early apoptosis: Annexin V^+^/PI^-^; late apoptosis: V^+^/PI^+^; necrotic: V^-^/PI^+^. **(E)** Caspase-3 activity of MSCs in all groups. Data are expressed as mean ± SEM. n = 5, *P <0.05, # *P* <0.05 versus H/SD. FACS, fluorescence-activated cell sorting; H/SD, hypoxia and serum deprivation; IP6Ks, inositol hexakis phosphate kinases; MSCs, mesenchymal stem cells; SEM, standard error of the mean; TNP, N6-(p-nitrobenzyl) purine; TUNEL, terminal- deoxynucleotidyl transferase mediated nick end labeling.

Representative TUNEL and flow cytometry results in Figure 5A,B demonstrated that H/SD significantly induced apoptosis of aged MSCs (36.73 ± 1.62% versus 16.67 ± 0.75% under normal conditions, *P* <0.05), while TNP pretreatment significantly decreased the percentage of apoptotic aged MSCs under normoxic and hypoxic conditions (11.53 ± 0.87% in normoxia and 25.63 ± 0.68% in H/SD, *P* <0.05).

### TNP enhanced paracrine efficiency of aged MSCs

**Figure 6:**
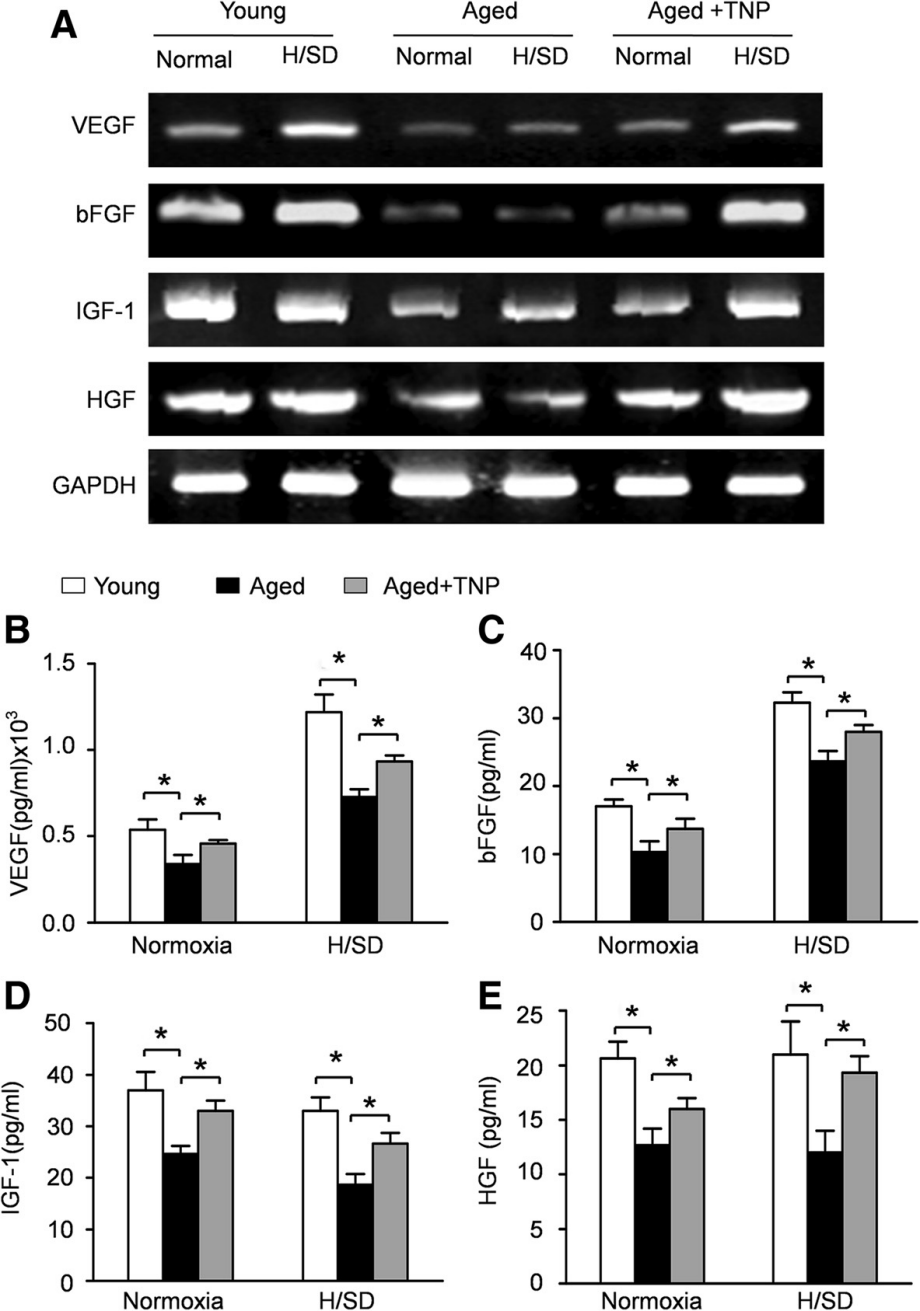
**TNP enhanced paracrine efficiency of aged MSCs. (A)** Representative RT-PCR results of the mRNA levels of VEGF, bFGF, IGF-1 and HGF in young MSCs, aged MSCs and even aged MSCs with TNP. The protein expressions of VEGF **(B)**, bFGF **(C)**, IGF-1** (D)** and HGF **(E)** were measured by ELISA assays. Data are expressed as mean ± SEM. n = 5, *P <0.05. bFGF, basic fibroblast growth factor; HGF, hepatocyte growth factor; IGF-1, insulin-like growth factor-1; MSCs, mesenchymal stem cells; SEM, standard error of the mean; TNP, N6-(p-nitrobenzyl) purine; VEGF, vascular endothelial growth factor.

It has been shown that MSCs contribute to cardiac repair and regeneration at least in part by a paracrine mechanism. Therefore, we performed RT-PCR and ELISA assays to evaluate the effect of aging on cytokine secretion in MSCs. We observed that young MSCs expressed VEGF ((0.49 ± 0.03) × 10^3^ pg/ml), bFGF (18.03 ± 1.57 pg/ml), IGF-1(37.21 ± 4.23 pg/ml) and HGF (21.28 ± 2.43 pg/ml) under normal conditions. After six hours H/SD injury, VEGF ((1.35 ± 0.08) × 10^3^ pg/ml) and bFGF (32.34 ± 2.15 pg/ml) secreted by young MSCs were significantly increased. Furthermore, RT–PCR (Figure 6A) and ELISA (Figure 6B-E) revealed that the expression (for both mRNA and protein levels) of growth factors, including VEGF, bFGF, IGF-1 and HGF, secreted by aged MSCs was significantly decreased in comparison to young MSCs, under both normoxic and hypoxic conditions. However, IP6KS inhibition with TNP enhanced the paracrine efficiency of aged MSCs under normoxic and hypoxic conditions.

## Discussion

Autologous bone marrow mesenchymal stem cells have been considered an ideal cell source for MI therapy [21]. A multitude of animal studies have demonstrated that MSCs could restore cardiac function [7]. However, intracoronary transplantation of autologous bone marrow stem cells led to slightly long-term myocardial functional improvement in our previous clinical study for STEMI patients after four years follow-up [5]. Given that a complex interplay between engrafted cells, cytokines and ischemic microenvironment may ultimately influence the clinical outcomes, BM-MSCs obtained from elderly patients seem less therapeutically efficacious for ischemic heart diseases [11]. In the present study, we demonstrated for the first time that MSCs from aged mice secreted significantly more IP7 compared to young mice, which leads to impaired Akt activation and paracrine effects. Furthermore, IP7 synthesis was also associated with the deterioration and impairment of aged MSCs in hypoxic conditions, indicating that age-related IP7 may play a critical role in the therapeutic efficacy of MSC for MI.

Aging is a complicated pathophysiological process which negatively impacts tissue repair and healing [22]. It has been reported that age influences the proliferation potential of MSCs [11]. Furthermore, the function and therapeutic potential of MSCs generally decline with age [10]. In the present study, we found that BM-MSCs from aged mice exhibit increased apoptosis compared with those from young mice. To further explore the influence of aging on the apoptosis of MSCs in an ischemic environment, we subjected cultured cells to hypoxia and serum deprivation conditions. Zhu *et al.* reported that the early apoptosis in MSCs induced by hypoxia and serum deprivation peaked at six hours [23]. Therefore, we also used the same model and found that the deleterious effects of aging appear to increase the apoptosis induced by H/SD injury, indicating that aging is associated with increased vulnerability to hypoxic injury in MSCs. Previous studies have demonstrated that hypoxia inhibits mitochondrial respiration and decreases mitochondrial DNA (mtDNA) genomes numbers, which in turn influence the mitochondrial permeability [24]. Loss of mitochondrial membrane potential results in the release of cytochrome c, which has been recognized as a regulator in apoptosis [25]. Apoptosis is a complex process which is modulated by extrinsic and intrinsic pathways [26]. Bcl-2 family proteins are the chief regulators which conduct the intrinsic pathways of apoptosis [27]. The family of BCL2 proteins, which consists of both anti-apoptosis proteins and pro-apoptosis proteins, is divided into three subgroups depending on the number of BH domains [28]. The pro-survival proteins, such as Bcl-2 and Bcl-xL, share four BH domains, whereas the pro-apoptotic proteins Bax and Bak contain three BH domains [29,30]. In the present study, we observed that the expressions of pro-apoptosis proteins, such as Bad and Bax, were significantly increased in aged MSCs under both normoxic and hypoxic conditions. Meanwhile, aging also decreased the expression of the anti-apoptosis protein Bcl-2. Furthermore, the inhibition of IP6Ks was associated with decreased BAD phosphorylation coupled with a decline in the Bax/Bcl-2 ratio. Taken together, these data indicate that aging regulates IP7 synthesis and the expression of BCL2 proteins which, in turn, mediate the apoptosis of MSCs.

Akt (PKB), a serine/threonine protein kinase, plays a central role in regulating cell survival, metabolism and protein synthesis via the phosphorylation of its numerous substrates. Given the multifunctional roles ascribed to Akt, it is likely that aging-related disorders are associated with Akt dysregulation. Watanabe *et al*. [31] demonstrated that impaired PI3K/Akt activation directly contributes to the effect of aging on pancreatic acinar cell proliferation. Similarly, Satyanarayana *et al.*[32,33] have revealed that aging-induced muscle atrophy is associated with differences in the regulation of Akt and mTOR. Furthermore, it has been reported that reduced activation of extracellular signal-regulated kinase (ERK) and Akt kinase contributes to lower survival of aged hepatocytes which are more sensitive to H_2_O_2_-induced apoptosis [34,35]. In the present study, we observed impaired activation of Akt in the MSCs. Furthermore, H/SD injury further reduced the aging-associated hyp-phosphorylation of Akt. These data indicate that the increased vulnerability of MSCs to hypoxic injury with aging is due to impaired Akt activation. Therefore, the restoration of Akt kinase activity has been considered as an attractive therapeutic target to protect aged MSCs from hypoxic injury. Various strategies have been adopted to enhance Akt activation in MSCs. Mangi *et al.*[36] genetically modified MSCs with Akt using retroviruses and found that the engineered MSCs (Akt-MSCs) were more resistant to hypoxic injury. Moreover, Haider *et al.*[30] revealed that MSCs with overexpression of IGF-1 display both increased Akt activation and enhanced viability. However, gene modifications might result in insertional mutagenesis. In addition, prolonged activation of constitutively Akt gene may increase the risk of tumorigenesis which hampers genetically engineered cells for clinical therapy [37]. An alternative strategy to gene delivery is temporary activation of Akt with pharmaceutical pretreatment.

Inositol polyphosphates, a diverse group of signaling molecules, are produced through the action of sequential phosphorylations by inositol polyphosphate kinases (IPKs) from IP3 [37]. IP3, which regulates intracellular calcium release, can be sequentially phosphorylated to generate inositol hexakisphosphate (IP6) and 5-diphospho-inositolpentakisphosphate (5-PP-IP5, or IP7). Although the physiological functions of IPs remain poorly characterized, IP7 appears to inhibit Akt signaling and to modulate cell apoptosis [17].

To explore the relationship between aging and IP7 level, we measured inositol phosphates in MSCs from both aged and young donor mice. The level of IP7 was elevated in aged mice, resulting in impaired Akt activation, which is consistent with the results of Chakraborty *et al.*[17]. Furthermore, hypoxic injury resulted in an enhancement in the age-dependent increase of IP7, indicating that increased IP7 with aging interferes with Akt activation. In addition, the rise in IP7 levels is substantially reduced by the IP6Ks inhibitor TNP, which was associated with decreased apoptosis and increased paracrine effect of MSCs, indicating that IP7 may be involved in the hypoxic injury of MSCs. Taken together, our results indicate that aging increases IP7 synthesis, which inhibits the activation of Akt signaling, leading to impairment after hypoxic injury.

MSCs are capable of secreting a broad variety of bioactive factors, such as VEGF, bFGF, HGF, and IGF-1, which enhance neovascularization and inhibit host cardiomyocyte death [7]. Therefore, a growing body of evidence supports the hypothesis that MSCs provide a beneficial effect through paracrine activity for clinical autologous cell therapy for MI. However, the effect of aging on the xparacrine activity of MSCs still remains unclear. We pursued our investigation further to elucidate aging dependent growth factor expression in MSCs. Previous studies have revealed that hypoxic stimulation could increase cytokine secretion in MSCs [7,11]. In our study, the gene expression of VEGF and bFGF in MSCs was significantly increased under hypoxic stress. Interestingly, no effect of hypoxia on the expressions of IGF-1 and HGF was observed. However, significant reductions in the secretion of angiogenic factors were observed in aged MSCs, especially under hypoxic conditions. These results are supported by the gene expression profiles, as determined by RT-PCR analyses. Certainly, our data suggest that aging negatively modulates the paracrine activity of MSCs. Furthermore, the inhibition of IP6Ks with TNP restored the decreased paracrine efficiency of aged MSCs under normoxic and hypoxic conditions. Therefore, our data indicate that aging impaired paracrine efficiency of MSCs at least in part via IP6Ks and IP7, which may be promising novel targets for augmenting the therapeutic efficacy of aged MSCs.

Although the H/SD model used in the present study is considered useful to exclude the influence of neural and humoral factors *in vivo*, this model is limited as an artificial experimental model that cannot fully simulate the *in vivo* ischemic and inflammatory environment. Moreover, the physiological functions of IP7 have not been extensively clarified. Therefore, further studies defining the exact mechanism(s) are needed to thoroughly understand the aging process of MSCs.

## Conclusions

In conclusion, the current study demonstrates that aging increases the vulnerability of MSCs to hypoxic injury with impaired paracrine efficiency. Moreover, increased IP7 formation by IP6Ks, which inhibits Akt activation, may directly contribute to the aging-related injury of MSCs. Furthermore, inhibition of IP6Ks has the potential to protect the aged MSCs via activation of Akt signaling. Overall, our results indicate that IP6Ks and IP7 may be promising novel modification targets for optimizing autologous MSCs therapy for MI.

## Abbreviations

bFGF: basic fibroblast growth factor; BM-MSCs: bone marrow-derived mesenchymal stem cells; DAPI: 4,6-diamidino-2-phenylindole; (D)MEM: (Dulbecco’s) modified Eagle’s medium; ELISA: enzyme-linked immunosorbent assay; FBS: fetal bovine serum; H/SD: hypoxia and serum deprivation; HGF: hepatocyte growth factor; HPLC: high performance liquid chromatography; IGF-1: insulin-like growth factor-1; IP3: inositol 1,4,5-trisphosphate; IP6Ks: inositol hexakis phosphate kinases; IP7: 5-diphosphoinositol pentakisphosphate; IPs: inositol phosphates; MI: myocardial infarction; MSCs: mesenchymal stem cells; PBS: phosphate-buffered saline; PI: propidium iodide; SDF-1α: stromal cell- derived factor-1α; TNP: N6-(p-nitrobenzyl) purine; TUNEL: terminal- deoxynucleotidyl transferase mediated nick end labeling; VEGF: vascular endothelial growth factor.

## Competing interests

The authors declare that they have no competing interests.

## Authors’ contributions

ZZ contributed to experimental design, performed all experiments, collected and interpreted data and wrote the manuscript. CXZ was involved in experimental design, data acquisition, data analysis and manuscript drafting. BL contributed to experimental design, isolation and characterization of MSCs, interpretation of data and manuscript drafting. DL was involved in experimental design, IP7 detection, data acquisition, data interpretation and manuscript drafting. XQ contributed to experimental design, western-blotting experiments, data acquisition, data analysis and manuscript drafting. XJL assisted in experimental design, provided apoptosis detection expertise and assisted in manuscript drafting. RQZ was involved in experimental design, assisted in isolation and characterization of MSCs and manuscript editing. CYL assisted in experimental design, provided western-blotting expertise and assisted in manuscript editing. HCW contributed to experimental design, IP7 detection, data interpretation and manuscript editing. DDS was involved in experimental design, assisted in western-blotting experiments, collected and interpreted data and wrote the manuscript. FC was involved in experimental design, performed all experiments, collected and interpreted data, supervised work and wrote the manuscript. All authors read and approved the final manuscript.
